# The impact of potential food allergen exposure on wound healing after malignant tumor surgery: immune tolerance alterations and clinical risk assessment

**DOI:** 10.3389/fimmu.2026.1873440

**Published:** 2026-08-03

**Authors:** Yingying Liu, Keyue Pan, Xueliang Zhang, Huanzhi Jin, Yue Lin, Zheyan Chen

**Affiliations:** 1 Medical Affairs Department, Wenzhou Third Clinical Institute Affiliated to Wenzhou Medical University, Wenzhou People’s Hospital, The Wenzhou People’s Hospital Affiliated of Hangzhou Medical College, Wenzhou, China; 2 Emergency Medicine Department, Wenzhou Third Clinical Institute Affiliated to Wenzhou Medical University, Wenzhou People’s Hospital, The Wenzhou People’s Hospital Affiliated of Hangzhou Medical College, Wenzhou, China; 3 Department of General Practice, Wenzhou Third Clinical Institute Affiliated to Wenzhou Medical University, Wenzhou People’s Hospital, The Wenzhou People’s Hospital Affiliated of Hangzhou Medical College, Wenzhou, China; 4 Department of Plastic and Reconstructive Surgery, Wenzhou Third Clinical Institute Affiliated to Wenzhou Medical University, Wenzhou People’s Hospital, The Wenzhou People’s Hospital Affiliated of Hangzhou Medical College, Wenzhou, China

**Keywords:** clinical risk, food allergens, immune tolerance, inflammatory response, post-malignant tumor surgery, wound healing

## Abstract

Wound healing after malignant tumor surgery is a critical determinant of patient recovery, and the dynamic changes in the postoperative immune system may alter tolerance to food allergens, thereby influencing the healing process. This review aims to systematically explore the alterations in immune tolerance following malignancy surgery, investigate the effects of potential food allergen exposure on wound healing, and analyze associated clinical risks. By integrating recent findings from immunology, nutrition, and surgery, we propose that postoperative allergen exposure may impede normal wound healing through mechanisms such as activating inflammatory responses, delaying angiogenesis, and disrupting collagen deposition. Additionally, this article discusses strategies for identifying high-risk patients, clinical management recommendations, and future research directions, providing evidence-based guidance for clinical practice.

## 1 Introduction

Surgical resection remains the cornerstone of curative-intent treatment for the majority of solid malignancies, including malignant melanoma, breast cancer, soft tissue sarcomas, and other solid tumors. Despite ongoing advancements in surgical techniques and perioperative care, postoperative complications—particularly impaired wound healing, surgical site infections (SSIs), and locoregional tumor recurrence—remain major clinical challenges that compromise patient prognosis, quality of life, and healthcare resource utilization. Malignant melanoma surgery, for instance, is frequently accompanied by significant cutaneous defects and challenges in wound closure; innovative strategies such as polypyrrole-modified hFGF2 oil bodies, sprayable therapeutic hydrogels, and peptide-based double-network hydrogels have been explored to simultaneously suppress tumor recurrence and promote wound healing through photothermal therapy, oxygen generation, or antibacterial and pro-angiogenic effects (1–3). Similarly, in breast cancer surgery, postoperative complications affect approximately one-third of patients and are associated with increased risks of disease recurrence and mortality (4). The potential tumor-promoting effects of the postoperative wound microenvironment are further supported by evidence that surgery-induced wound fluid contains bioactive factors capable of promoting breast cancer cell proliferation, spheroid formation, and malignancy-associated phenotypes in 2D and 3D culture models (5, 6). Furthermore, radiotherapy-induced remodeling of the tumor microenvironment may influence tumor cell behavior and further complicate postoperative tumor–microenvironment interactions (7). Together, these observations indicate that the postoperative period represents an active biological phase characterized by substantial remodeling of local and systemic immune environments. Following surgical trauma, a complex inflammatory cascade is initiated, characterized by acute-phase protein release, a cytokine surge, and the recruitment of innate and adaptive immune cells to the wound site. This response, while essential for tissue repair, also creates a microenvironment that can paradoxically promote the survival and proliferation of residual tumor cells (8–10). Proteomic profiling of healthy living kidney donors undergoing laparoscopic nephrectomy has revealed significant perioperative upregulation of acute-phase proteins, including C-reactive protein (CRP), serum amyloid A1 (SAA1), serum amyloid A2 (SAA2), and lipopolysaccharide-binding protein and tissue repair factors such as fibrinogen-like protein 1 and alpha-2-glycoprotein 1, indicating that even in otherwise healthy individuals, laparoscopic surgery can induce a measurable systemic stress response (11). Beyond these inflammatory effects, emerging evidence from glioma studies suggests that surgery may act as an evolutionary bottleneck by reshaping tumor evolution through selective pressures that favor the expansion of invasive and stem-like tumor cell subpopulations, thereby contributing to clonal selection and molecular divergence at recurrence (12). The consequences of impaired wound healing transcend localized sequelae. Delayed closure of surgical wounds creates a conduit for microbial invasion, thereby substantially elevating the risk of SSIs and periprosthetic joint infections in orthopedic oncology. Data from the nationwide bone tumor registry in Japan indicate that the incidence of SSI/periprosthetic joint infection following tumor endoprosthesis reconstruction is 8.2%, with rates as high as 41.2% for pelvic reconstructions and 12.6% for proximal tibia cases; delayed wound healing was identified as an independent risk factor for infection (13). Moreover, the presence of residual tumor cells in the wound bed, compounded by the growth-promoting and the immunosuppressive effects of the healing microenvironment, establishes a self-perpetuating cycle that heightens the risk of local recurrence. Biopsy procedures prior to definitive surgery for proximal fibular tumors, for example, have been associated with a 7.6-fold greater risk of impaired wound healing, underscoring the delicate interplay between diagnostic interventions, surgical trauma, and postoperative outcomes (14). Despite these well-documented challenges, the potential impact of food allergen exposure on the wound healing process and the evolving tumor immune microenvironment remains a potentially important yet largely unexplored consideration in the postoperative period. Although direct evidence linking postoperative dietary allergen exposure with impaired wound healing remains unavailable, emerging insights into immune tolerance, allergic inflammation, and tissue repair pathways suggest plausible mechanistic connections that warrant further investigation. The immune system’s capacity to maintain oral tolerance to dietary antigens is a tightly regulated process, governed by the activity of regulatory T cells and tolerogenic dendritic cells and the integrity of the gut-associated lymphoid tissue. Major surgery, with its associated stress response, systemic inflammation, and immune dysregulation, may disrupt this tolerance, predisposing patients to developing aberrant immune responses following exposure to common allergens such as milk, eggs, peanuts, or soy. While the direct association between postoperative food allergy and wound healing has not been extensively studied, the immunological parallels between allergic inflammation and wound repair suggest plausible mechanistic links. Allergic reactions to food allergens are mediated by T helper 2 (Th2)-type immune responses, characterized by the production of IL-4, IL-5, and IL-13, which promote eosinophil recruitment, mast cell degranulation, and immunoglobulin E (IgE) class switching. Although these processes are traditionally linked to atopic disease, they also converge on pathways critical for wound healing, including macrophage polarization, angiogenesis, and ECM remodeling. Th2-skewed immune environments have been implicated in both fibrosis development and the suppression of anti-tumor immunity. IL-33, a key regulator of type 2 immunity released following tissue damage, has been implicated in shaping the tumor microenvironment and may contribute to immune evasion and tumor progression (15). This dual role of tissue repair pathways in tumor progression is reflected in the concept of the tumor microenvironment as “a wound that never heals”. Tumors co-opt the wound healing machinery to support their growth and dissemination, as evidenced by how breast cancer cells hijack hyaluronan signaling pathways—normally involved in the balance between pro- and anti-inflammatory responses during tissue repair—to drive invasion and immune avoidance (16). Accordingly, the introduction of a food allergen into an already fragile and dysregulated immune environment could theoretically tilt the balance further toward immune suppression or inappropriate inflammation, with adverse consequences for both wound closure and tumor control. The clinical relevance of this hypothesis is supported by the growing recognition that dietary factors can influence cancer management and postoperative recovery. Enhanced recovery after surgery (ERAS) protocols now emphasize early enteral nutrition (EN) to support gastrointestinal function, immune competence, and metabolic recovery; however, the immunological implications of specific dietary components—including potential allergens—during the immediate postoperative period remain poorly defined. A study of early EN under the ERAS model in patients undergoing gastric tumor surgery demonstrated significant improvements in the recovery of gastrointestinal and immune function, as evidenced by higher serum levels of immunoglobulins (IgG, IgM, IgA) and reduced inflammatory markers compared to parenteral nutrition alone (17). While these findings support the benefits of early enteral feeding, they also raise the question of whether the type of nutrients provided, particularly those with allergenic potential, could modulate immune recovery and influence postoperative complications. Moreover, evidence from injury models indicates that soluble factors present in tissue damage-associated fluids can actively shape macrophage polarization and influence tumor-related immune responses. For instance, burn wound exudates have been shown to modulate macrophage polarization and alter macrophage-mediated anti-tumor activity, suggesting that injury-associated immune environments may affect tumor–immune interactions (18). The plasticity of macrophages in response to environmental cues—including those present in both wound and tumor microenvironments—suggests that dietary antigens could similarly influence the local immune contexture. These findings suggest that the wound healing environment is sensitive to exogenous immune mediators, raising the possibility that dietary allergens may influence local immune contexture and postoperative immune regulation. Finally, the potential for food allergen exposure to influence the tumor microenvironment must be considered within the broader framework of immune checkpoint biology and precision oncology. The tumor microenvironment in many cancers, including melanoma, breast cancer, and pancreatic ductal adenocarcinoma, is characterized by immune suppression mediated by regulatory T cells, alternatively activated (M2) macrophages, and myeloid-derived suppressor cells (19, 20). Factors such as transforming growth factor-beta (TGF-β) and vascular endothelial growth factor (VEGF), which are upregulated in the wound healing context, also drive the innate anti-PD-1 resistance signatures associated with poor response to immunotherapy (19). As allergic inflammation can either promote or suppress anti-tumor immunity depending on the context, defining how food antigens interact with the postoperative immune milieu may provide new insights into risk assessment and nutritional management. This review examines the emerging interplay between postoperative food allergen exposure, immune tolerance dynamics, and wound healing in the context of malignant tumor surgery. By synthesizing evidence from studies on surgical trauma, wound healing, tumor immunology, and immune tolerance to dietary antigens, this review proposes potential mechanisms by which allergen exposure may influence postoperative recovery and long-term oncological outcomes, while outlining a framework for clinical risk assessment and personalized nutritional management. Although several biological mechanisms discussed in this review are supported by experimental and translational studies, direct clinical evidence linking postoperative food allergen exposure to impaired wound healing in cancer patients remains limited. Therefore, where appropriate, established observations are distinguished from hypothesis-generating concepts, and proposed mechanisms should be interpreted within the context of the currently available evidence.

## 2 Literature search strategy

We searched PubMed, Web of Science, Embase, and Scopus databases from database inception to June 2026 using combinations of the following terms: “food allergy”, “food allergen”, “IgE-mediated allergy”, “oral tolerance”, “imne tolerance”, “regulatory T cells”, “macrophage polarization”, “wound healing”, “tissue repair”, “surgical trauma”, “oncologic surgery”, “cancer surgery”, “tumor microenvironment”, and “postoperative inflammation”. Original research articles, clinical studies, translational investigations, and relevant review articles were considered, with priority given to studies addressing immune regulation, allergic inflammation, wound-healing biology, and postoperative immune alterations associated with cancer surgery. When direct clinical evidence was limited, mechanistic studies from related fields were also included.

## 3 Remodeling of the postoperative immune microenvironment in malignant tumors

### 3.1 Surgical trauma and the acute inflammatory response

The initiation of an acute inflammatory response is an early consequence of surgical trauma, as tissue injury activates the innate immune system through the release of damage-associated molecular patterns (DAMPs). These endogenous danger signals, including high-mobility group box 1, activate pattern recognition receptors such as Toll-like receptors, thereby triggering sterile inflammatory cascades that promote the release of pro-inflammatory cytokines, including interleukin (IL)-1β, tumor necrosis factor-alpha (TNF-α), and IL-6 (21). This local inflammatory reaction rapidly progresses into a systemic stress response, involving neuroendocrine activation, immune modulation, and the acute-phase response (APR) (22). For instance, clinical studies and animal models have shown that major surgery induces significant post-operative increases in acute-phase proteins such as C-reactive protein and SAA (23–25). The magnitude of this response is influenced by the extent of surgical trauma, with more invasive procedures generally associated with greater and more sustained inflammatory activation compared with less invasive approaches (26, 27).

This post-surgical inflammatory state can profoundly alter intestinal barrier function, potentially increasing exposure to luminal antigens, including dietary antigens. Systemic inflammatory cytokines, including TNF-α and IL-1β, can compromise intestinal epithelial integrity by disrupting tight junction proteins and increase gut permeability (28). This disruption may allow macromolecules, including dietary antigens that are normally restricted by the gut barrier, to translocate across the intestinal wall and enter the systemic circulation. Experimental models of polytrauma and hemorrhagic shock have demonstrated that trauma-induced inflammation is associated with increased intestinal permeability and disruption of tight junction proteins, including zonula occludens-1 and occludin (29). Consequently, the immune system of postoperative patients is exposed to an increased load of dietary antigens, potentially predisposing them to aberrant immune responses.

Critically, the “inflammatory storm” peaking during the early postoperative period (24–72 hours) can impair the function of regulatory T cells (Tregs), vital for maintaining peripheral tolerance and preventing allergic responses. Elevated IL-6 levels can impair Treg differentiation and suppressive function, potentially disrupting oral tolerance to dietary antigens (30). Beyond barrier disruption, postoperative inflammatory signals may further influence adaptive immune regulation by altering the balance between regulatory and effector T-cell responses, with altered Treg frequency and function, together with impaired effector T-cell activity, contributing to postoperative immune dysfunction (31, 32). This functional impairment of Tregs during the critical perioperative window may compromise the establishment and maintenance of oral tolerance to newly encountered food antigens. Thus, surgical trauma-induced inflammation may simultaneously increase antigen exposure through gut barrier disruption and weaken Treg-mediated regulation, reducing tolerance toward newly encountered antigens.

### 3.2 Persistent impact of tumor-associated immunosuppression

Postoperative cellular immune suppression is most pronounced during the early postoperative period, typically reaching its maximum within the first several days after surgery. Although immune function gradually recovers thereafter, residual alterations in both innate and adaptive immune responses may persist for several weeks in some patients, depending on the extent of surgical trauma, tumor burden, and individual immune status (33, 34). Postoperative immunosuppression is characterized by the accumulation of various suppressive myeloid populations, including myeloid-derived suppressor cells (MDSCs) and tumor-associated macrophages (TAMs), as exemplified by the identification of CX3C chemokine receptor 1^+^ (CX3CR1^+^) immunosuppressive myeloid cells in the peritoneal cavity after gastric cancer resection (35). These cells potently inhibit anti-tumor immunity by suppressing T cell activation, impairing antigen presentation by dendritic cells, and secreting anti-inflammatory cytokines like IL-10 and TGF-β (26). Surgical intervention, while removing the bulk of the tumor, does not immediately reverse this pre-existing global immune dysfunction. Furthermore, the additional trauma of surgery may further exacerbate immunosuppressive pathways, as the postoperative inflammatory response co-occurs with a compensatory anti-inflammatory response syndrome a state that is often particularly pronounced in cancer patients (26). Postoperative immune dysregulation may compromise the mechanisms responsible for maintaining oral immune tolerance through altered antigen presentation, impaired regulatory T-cell function, and disruption of mucosal immune homeostasis, thereby increasing susceptibility to inappropriate immune responses against dietary antigens in susceptible individuals (31, 32).

The prolonged postoperative immunosuppression may compromise the processes of antigen recognition and tolerance induction, which are fundamental for a safe immune response to food proteins. Effective oral tolerance relies on the immune system’s ability to recognize food antigens in the gut and generate Tregs in a non-inflammatory context. However, in the post-surgical cancer patient, antigen-presenting cell (APC) function may be compromised by immunosuppressive populations, including MDSCs and TAMs—a scenario that is plausible given the persistent immunosuppressive milieu documented in the postoperative period, such as the accumulation of CX3CR1+ immunosuppressive cells after gastric cancer resection (35). This impaired APC function may hamper the efficient presentation of food antigens to naïve T cells in a tolerogenic manner. Instead, the prevailing inflammatory and immunosuppressive signals—which are associated with a shift in the T helper 1 (Th1)/Th2 balance toward Th2—may favor the differentiation of these T cells toward an effector phenotype that is more prone to producing pro-allergic cytokines (36). The immune system’s failure to appropriately recognize and process these new antigens in a tolerogenic manner during this vulnerable period may contribute to the impairment of establishing robust oral tolerance.

This state of immune dysregulation may create a scenario whereby exposure to food allergens triggers an atypical and potentially allergic immune response rather than tolerance. Instead of the default induction of Tregs, the combination of a Th2-skewed immune environment reported in certain cancers and impaired APC function may lead to the activation of allergen-specific Th2 cells that produce IL-4, IL-5, and IL-13 (37). IL-4 and IL-13 are key cytokines involved in regulating B-cell class switching toward IgE production and the development of pathogenic IgE-producing B-cell responses (38, 39), whereas IL-5 contributes to the maintenance of chronic IgE responses and the differentiation of antibody-secreting cells in inflamed tissues (40). A study following gastric cancer surgery identified that CX3C chemokine receptor 1+ cells contribute to an immunosuppressive environment in the peritoneal cavity that is associated with a higher rate of recurrence, indicating that the tumor-driven immunosuppressive networks persist and are active in the perioperative period (35). These data suggest that tumor-associated immunosuppressive networks may remain active after surgery, creating a milieu that favors a Th2-skewed immune response at the expense of tolerance, thereby increasing the risk of *de novo* sensitization to food allergens or exacerbation of pre-existing allergic conditions.

### 3.3 Immunomodulatory effects of anesthesia and perioperative medications

The pharmacologic agents administered during the perioperative period—including anesthetics, analgesics, and antibiotics—exert significant immunomodulatory effects that can further disrupt the delicate balance of the immune system and may contribute to conditions that favor allergic sensitization. Inhaled anesthetics, such as sevoflurane, and opioid analgesics have been demonstrated to directly suppress key components of the cellular immune response. For example, sevoflurane has been shown to decrease natural killer (NK) cell activity and suppress T-cell proliferation (41), while major surgery combined with general anesthesia can impose substantial immunological stress during the perioperative period (42). Opioids exert complex immunomodulatory effects that extend beyond simple immune suppression. Depending on the specific compound and clinical context, opioid exposure may influence both innate and adaptive immune responses through mechanisms that can either suppress or modulate inflammatory signaling. Therefore, the overall immunological consequences of perioperative opioid administration are likely to depend on multiple clinical and pharmacological factors rather than a uniformly immunosuppressive effect (43–45). The cumulative effect of these agents is a temporary but substantial suppression of immune surveillance during and immediately after surgery, a period when the body is already dealing with the systemic inflammatory insult of the surgical wound (42, 46).

The use of glucocorticoids and broad-spectrum antibiotics during the perioperative period can further disrupt immune homeostasis through distinct mechanisms. Antibiotics primarily affect the composition and function of the gut microbiota, whereas glucocorticoids may influence intestinal barrier function and mucosal immunity. Intestinal microbes play a critical and non-redundant role in the induction and maintenance of oral tolerance by educating the host immune system to distinguish between harmless food antigens and pathogens. Perioperative antibiotics can disrupt the diversity and reduce the abundance of certain commensal bacteria, creating a state of dysbiosis (47). This microbial disruption can impair the generation of Tregs in the gut-associated lymphoid tissue (GALT), a key mechanism for preventing inappropriate immune responses to food antigens. While perioperative glucocorticoids are widely administered because of their anti-inflammatory and antiemetic effects, preclinical evidence suggests that they may also modulate intestinal barrier function and mucosal immune homeostasis (48). Whether these immunomodulatory effects alter the induction or maintenance of oral tolerance during the postoperative period remains uncertain, and direct clinical evidence in patients undergoing cancer surgery is currently limited. The combination of these drug classes therefore may compromise the integrity of the gut ecosystem, a system that is central to safeguarding the host against the development of food allergies.

**Figure 1:**
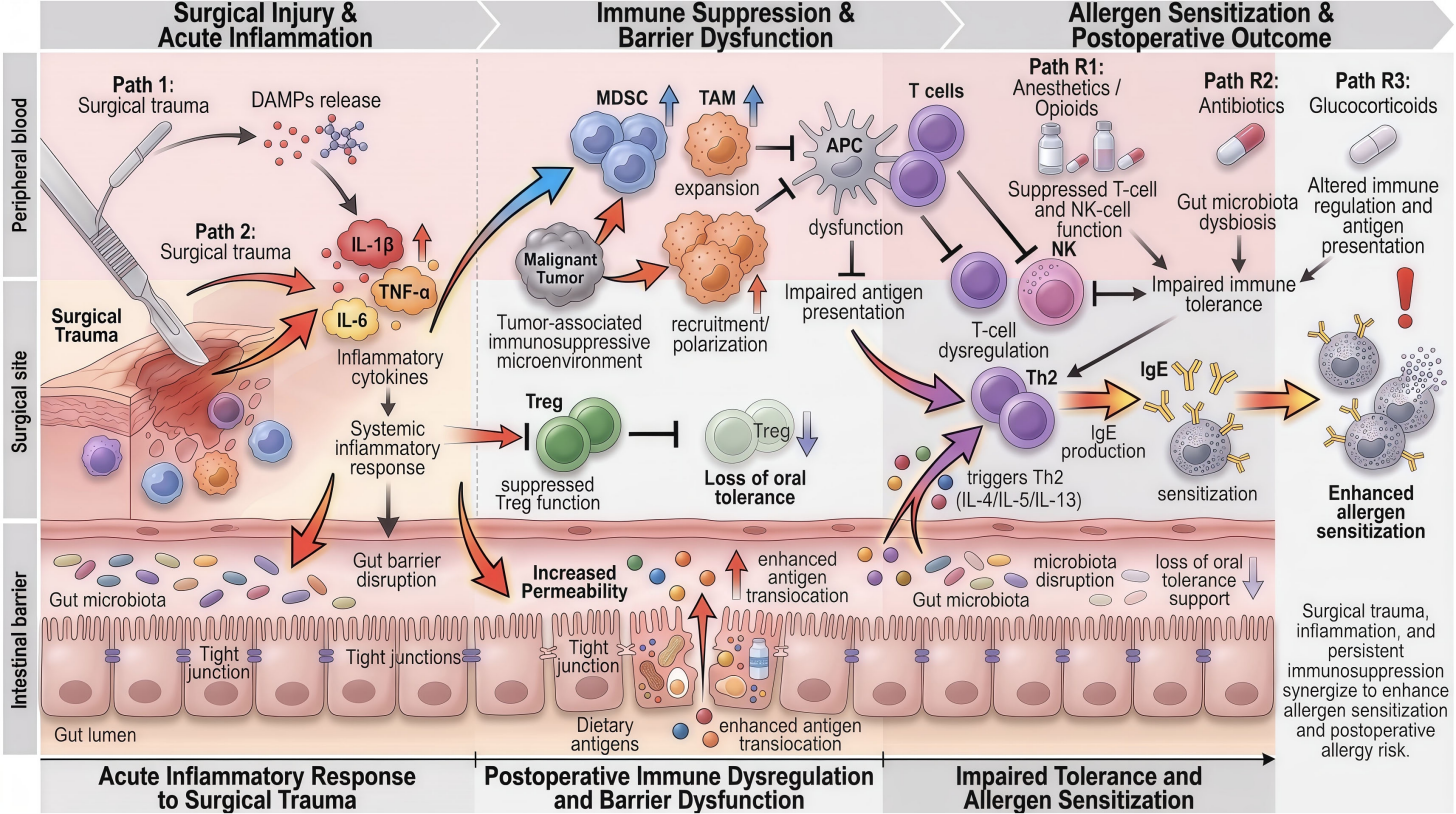
Remodeling of the postoperative immune microenvironment and potential mechanisms promoting food allergen sensitization after cancer surgery. This schematic summarizes the proposed progression from surgical inflammation to postoperative immune dysregulation and subsequent food allergen sensitization. Surgical trauma triggers DAMP release and sterile inflammatory cascades, contributing to systemic inflammation characterized by elevated IL-1β, TNF-α, and IL-6. Pro-inflammatory signaling compromises intestinal tight junction integrity, increasing permeability and facilitating dietary antigen translocation, while disrupting Treg-dependent immune homeostasis and oral tolerance. Tumor-derived immunosuppression may persist postoperatively through myeloid-derived suppressor cells (MDSCs) and tumor-associated macrophages (TAMs); these suppressive populations impair antigen presentation and promote a Th2-biased immune environment. Under these postoperative immune conditions, translocated luminal antigens may elicit allergen-specific Th2 responses characterized by IL-4, IL-5, and IL-13 production, thereby promoting B-cell IgE class switching and subsequent allergic sensitization. Perioperative medications, including anesthetics and opioids, antibiotics, and glucocorticoids, may further exacerbate immune dysfunction via distinct pathways: anesthetics and opioids suppress T- and NK-cell activity; antibiotics disrupt gut microbial homeostasis and gut-associated immune tolerance; glucocorticoids interfere with systemic immune signaling and antigen presentation. Collectively, these cumulative perioperative insults may create a transient period of increased immune susceptibility that compromises oral tolerance and increases the risk of *de novo* food allergen sensitization.

These pharmacological agents, through their immunomodulatory effects and antibiotic-associated gut dysbiosis, may create a vulnerable “window of opportunity” for allergen sensitization in the postoperative period. The transient suppression of T cell and NK cell activity by anesthetics and opioids, combined with antibiotic-associated disruption of the tolerogenic microbiota, may weaken immunological barriers that normally limit allergen sensitization (41, 47, 49). During this critical window, newly encountered or previously tolerated food allergens that have entered the circulation due to surgery-induced gut barrier dysfunction can be presented to an improperly programmed immune system (50, 51). The meta-analysis showing that combined general and epidural anesthesia is associated with a diminished postoperative inflammatory response compared to general anesthesia alone suggests that anesthetic technique may influence perioperative immune regulation. However, the available evidence does not support attributing this effect solely to reduced perioperative opioid exposure, and multiple mechanisms are likely to contribute to the observed immunological differences (52, 53). This finding underscores the potential for drug-induced immune modulation to alter clinical outcomes, potentially including the risk of postoperative allergic reactions. By opening this window of susceptibility, the very medications designed to ensure a safe and pain-free surgery may inadvertently increase the patient’s risk of developing new allergies (54). The potential mechanisms by which surgery-induced immune dysregulation may promote food allergen sensitization, as discussed above, are summarized in **Figure 1**.

## 4 Mechanisms of food allergen exposure impact on wound healing

### 4.1 Allergen-induced inflammatory response and the wound microenvironment

Exposure to potential food allergens in the postoperative context can fundamentally disrupt the wound healing process by initiating a robust inflammatory cascade that directly impairs the wound microenvironment. The initial and most immediate consequence of allergen-specific IgE crosslinking on the surface of dermal mast cells is degranulation, a process that releases a potent cocktail of preformed and newly synthesized inflammatory mediators (55, 56). The released key mediators, such as histamine and leukotrienes, exert profound effects on the local microvasculature. Histamine, for instance, directly is thought to contribute to vasodilation and significantly increases vascular permeability by inducing the contraction of endothelial cells and the formation of intercellular gaps (57, 58). This may contribute to plasma extravasation and the accumulation of fluid in the interstitial space, manifesting clinically as localized edema and swelling. While a degree of inflammation is necessary for the initial debridement phase of wound healing, the excessive and persistent edema driven by an allergic response can severely compromise the wound microenvironment (59, 60). Excessive edema can increase the diffusion distance between capillaries and surrounding tissues, impair local perfusion, and limit oxygen and nutrient availability, thereby creating an unfavorable environment for cellular proliferation and tissue repair (61). The edema fluid itself contains a milieu of inflammatory proteins and enzymes that can further damage the fragile ECM of the healing wound bed, creating a hostile environment for the ingress of reparative cells.

Beyond the acute effects of degranulation, the allergic response is characterized by a polarized T-helper 2 (Th2) inflammatory profile, dominated by cytokines such as IL-4 and IL-13. This Th2-biased immune environment may interfere with the coordinated functional transition of macrophages, which play essential roles throughout the wound-healing process. During normal tissue repair, macrophages initially exhibit predominantly classically activated (M1-like) phenotype to facilitate pathogen clearance and debris removal, followed by a transition toward an M2-like phenotype that supports tissue remodeling and resolution of inflammation (62, 63). M2 macrophages are the primary source of key growth factors, including TGF-β, which is critical for collagen synthesis and scar formation, and VEGF, which drives angiogenesis. Sustained IL-4 and IL-13 signaling may skew macrophage activation toward a persistent type 2 immune phenotype that differs from the temporally regulated reparative macrophage program required for effective wound healing. This dysregulated macrophage activation can lead to a state of macrophage dysfunction, characterized by a diminished capacity to secrete the necessary growth factors for tissue regeneration (64, 65). Consequently, the wound remains arrested in a chronic, non-healing inflammatory state, characterized by a deficiency in the necessary signaling molecules to progress to the proliferative phase. The failure to generate sufficient TGF-β and VEGF directly deprives the wound microenvironment of the signals needed for fibroblast activity and angiogenesis, stalling the healing process (66, 67).

Furthermore, the allergic inflammatory milieu is a potent driver of the expression and activity of matrix metalloproteinases (MMPs), a family of enzymes responsible for ECM degradation. Inflammatory mediators, including lipid-derived signals such as prostaglandin E2, together with cytokine-driven pathways, may contribute to the dysregulation of protease activity in chronically inflamed tissues by upregulating matrix metalloproteinase-9 (MMP-9) expression through EP2/EP4 receptor-mediated signaling (68). Excessive activation of matrix metalloproteinases (MMPs), particularly MMP-2 and MMP-9, disrupts extracellular matrix remodeling and contributes to impaired wound healing (69, 70). While MMP activity is essential for the normal remodeling of the ECM during wound healing, excessive and unregulated MMP activity, as seen in chronic inflammatory states, has been proposed to a net degradation of collagen and other ECM components. This accelerated collagenolysis overwhelms the synthetic capacity of fibroblasts, leading to a fragile, non-cohesive wound matrix. The resulting imbalance, where the rate of ECM destruction far exceeds the rate of new deposition, is a hallmark of chronic non-healing wounds. As discussed above, a dysregulated Th2-dominant immune environment may contribute to persistent inflammatory activation, aberrant tissue remodeling, and impaired tissue repair (71, 72). In summary, the allergic response initiates a vicious cycle in which mediator-induced edema and a persistent Th2-driven inflammation not only physically compress and compromise wound perfusion but also reprogram key immune cells to a dysfunctional state and release catabolic enzymes that directly dismantle the newly forming tissue scaffold. This multifactorial disruption of the wound microenvironment creates a formidable barrier to successful healing, increasing the risk of infection and the development of chronic wounds.

### 4.2 Impairments in angiogenesis and tissue oxygenation

The successful healing of any wound is critically dependent on the timely formation of new blood vessels, a process known as angiogenesis, to restore adequate perfusion, oxygenation, and nutrient delivery to the regenerating tissue. Allergen exposure may disrupt angiogenic processes via inflammatory mediators implicated in endothelial injury and aberrant vascular remodeling. A key effector cell in the allergic response, the eosinophil, degranulates upon activation and releases a suite of highly cytotoxic proteins, including major basic protein (MBP) and eosinophil cationic protein (ECP). These proteins are not merely inflammatory markers; they possess potent direct cytotoxic activity. Eosinophil-derived cytotoxic proteins, including MBP and ECP, have been reported to induce endothelial injury and vascular dysfunction in inflammatory diseases, suggesting that eosinophil activation may negatively influence the vascular remodeling processes required for tissue repair. This toxic assault can lead to endothelial cell apoptosis, detachment from the basement membrane, and a loss of vascular integrity (73). The resulting microvascular injury not only may influence further edema and micro-hemorrhage but, more critically, destroys the very cellular substrate necessary for the sprouting of new capillaries. The intact, healthy endothelium of existing blood vessels is the source from which new vessels are expected to grow through a process of proliferation and migration. However, eosinophil-derived mediators may impair endothelial integrity and functional competence, thereby compromising the vascular remodeling capacity required for efficient angiogenesis. This damage is distinct from the vasodilation caused by histamine and represents a more profound and long-lasting structural injury to the vascular network.

At the molecular level, allergic inflammatory signaling may interfere with the hypoxia-adaptive function of hypoxia-inducible factor-1 alpha (HIF-1α). In a normal wound, the local tissue environment is profoundly hypoxic due to disrupted vasculature. This hypoxia is a critical physiological signal that stabilizes the HIF-1α protein, preventing its degradation. Stabilized HIF-1α then translocates to the nucleus, where it acts as a master transcription factor, upregulating a large battery of genes essential for survival in low-oxygen conditions. Most prominently among these is VEGF, a key pro-angiogenic mediator that drives endothelial proliferation, migration, and new vessel formation (74). Persistent allergic inflammation may further disrupt the hypoxia-adaptive response within the wound microenvironment through sustained production of pro-inflammatory cytokines and oxidative stress. Such inflammatory alterations may affect HIF-1α signaling, which is closely coupled with inflammatory and oxidative stress pathways during chronic inflammation (75). Reduced HIF-1α activity may subsequently impair VEGF-mediated endothelial proliferation, migration, and new vessel formation during wound repair (76, 77). This dysregulation may result in insufficient VEGF induction despite persistent tissue hypoxia. Without adequate VEGF production, endothelial cells do not receive the necessary signals to proliferate, migrate, and organize into new tubular structures. Consequently, the hypoxia-driven angiogenic response may fail to achieve adequate activation, limiting the formation of functional granulation tissue.

The combined effect of endothelial cell destruction and the suppression of key angiogenic growth factors creates a state of severe and persistent tissue hypoxia within the wound. This persistent hypoxic state has several detrimental consequences for wound repair. First, reduced oxygen availability may impair the function of key reparative cells in the wound, including fibroblasts and keratinocytes, thereby limiting collagen production, extracellular matrix remodeling, cell proliferation, and re-epithelialization (78, 79). Second, and critically, oxygen is essential for effective antimicrobial activity by neutrophils and macrophages. Neutrophils depend on oxygen-dependent antimicrobial mechanisms, including respiratory burst–mediated generation of reactive oxygen species such as superoxide, to eliminate invading pathogens (80). A hypoxic wound may compromise this essential immune defense, thereby increasing the risk of infection (81). This is particularly dangerous in a postoperative setting where surgical wounds are already vulnerable. Third, reduced oxygen availability favors the colonization and persistence of anaerobic and facultative anaerobic bacteria within the wound environment (82). These infections are often difficult to treat and create an environment rich in bacterial byproducts and toxins that further destroy tissue and amplify the inflammatory response. This establishes a self-amplifying feedback loop: allergen exposure may impair angiogenesis, causing hypoxia; hypoxia compromises immune clearance of bacteria, leading to infection; the infection-induced inflammation may in turn exacerbate local tissue damage and further disrupt angiogenic signaling, including VEGF-related pathways, potentially perpetuating the hypoxic microenvironment. This cycle of poor vascularization, oxygen deprivation, and unchecked infection may contribute to delayed wound healing, wound dehiscence, and progression toward chronic wound states, thereby increasing patient morbidity.

### 4.3 Abnormalities in collagen deposition and scar remodeling

The final and most clinically visible phase of wound healing involves the deposition of collagen to restore tissue integrity and the subsequent remodeling of this scar tissue over months to years. The allergic inflammatory milieu, however, can profoundly derail both the quantity and quality of collagen, leading to either fragile, weak wounds or disfiguring, pathological scars. Th2-associated cytokines, including IL-5 and IL-9, have been associated with fibrotic remodeling in allergic diseases and may contribute to altered fibroblast activity through cytokine-mediated signaling pathways (83, 84). While this can initially lead to a state of heightened fibroblast proliferation and increased collagen production, the critical issue is the resulting imbalance in collagen subtypes. Normal dermis and mature scar tissue are composed predominantly of type I collagen, which forms large, cross-linked fibrils that provide tensile strength. During early wound healing, a provisional matrix rich in type III collagen, which forms thinner, less organized fibrils, is laid down. As healing progresses, type III collagen should be gradually replaced by type I collagen through a process of remodeling (85). Under the influence of the allergic response, this transition is faulty. Persistent Th2-driven signaling may disturb this collagen transition, potentially favoring excessive type III collagen deposition and impaired scar maturation. This has been proposed to a scar that is mechanically weaker, more fragile, and prone to tearing or re-injury. This is particularly problematic after surgery, where the wound needs to withstand significant mechanical stress. Alternatively, in some cases, the unbridled stimulation of fibroblasts can lead to an overproduction of both collagen types, coupled with a lack of the normal signals that regulate fibroblast apoptosis, culminating in hypertrophic scars or keloids that are raised, thickened, and often pruritic or painful (86, 87).

The orchestration of scar formation and wound contraction is critically dependent on the activity of the cytokine TGF-β. TGF-β is a master regulator of the healing process, responsible for recruiting fibroblasts, stimulating collagen synthesis, and, most importantly, inducing the differentiation of fibroblasts into myofibroblasts. Myofibroblasts are specialized contractile cells that express alpha-smooth muscle actin and are the primary drivers of wound contraction, pulling the wound edges together to close the defect (87). The allergic immune response creates a complex and often contradictory signaling environment for TGF-β. On one hand, the chronic inflammation can lead to sustained activation of some TGF-β-dependent pathways, contributing to the fibrosis seen in the abnormal collagen deposition described above. On the other hand, the presence of Th2-associated cytokines like IL-13 can directly alter the downstream signaling of TGF-β. For instance, IL-13 signaling through signal transducer and activator of transcription 6 has been implicated in fibroblast activation, extracellular matrix production, and fibrotic remodeling in pathological settings such as keloid formation (88). The result is a dysfunctional state where fibroblasts may produce excessive collagen but lack the contractile machinery needed to close the wound effectively. This disruption of myofibroblast differentiation can lead to a wound that remains open, with poor approximation of the wound edges. Wound dehiscence, the partial or total separation of the wound layers, is a severe postoperative complication that may be associated with impaired myofibroblast-mediated contraction (89, 90). This not only delays healing but also creates a wide, unsightly scar.

**Figure 2:**
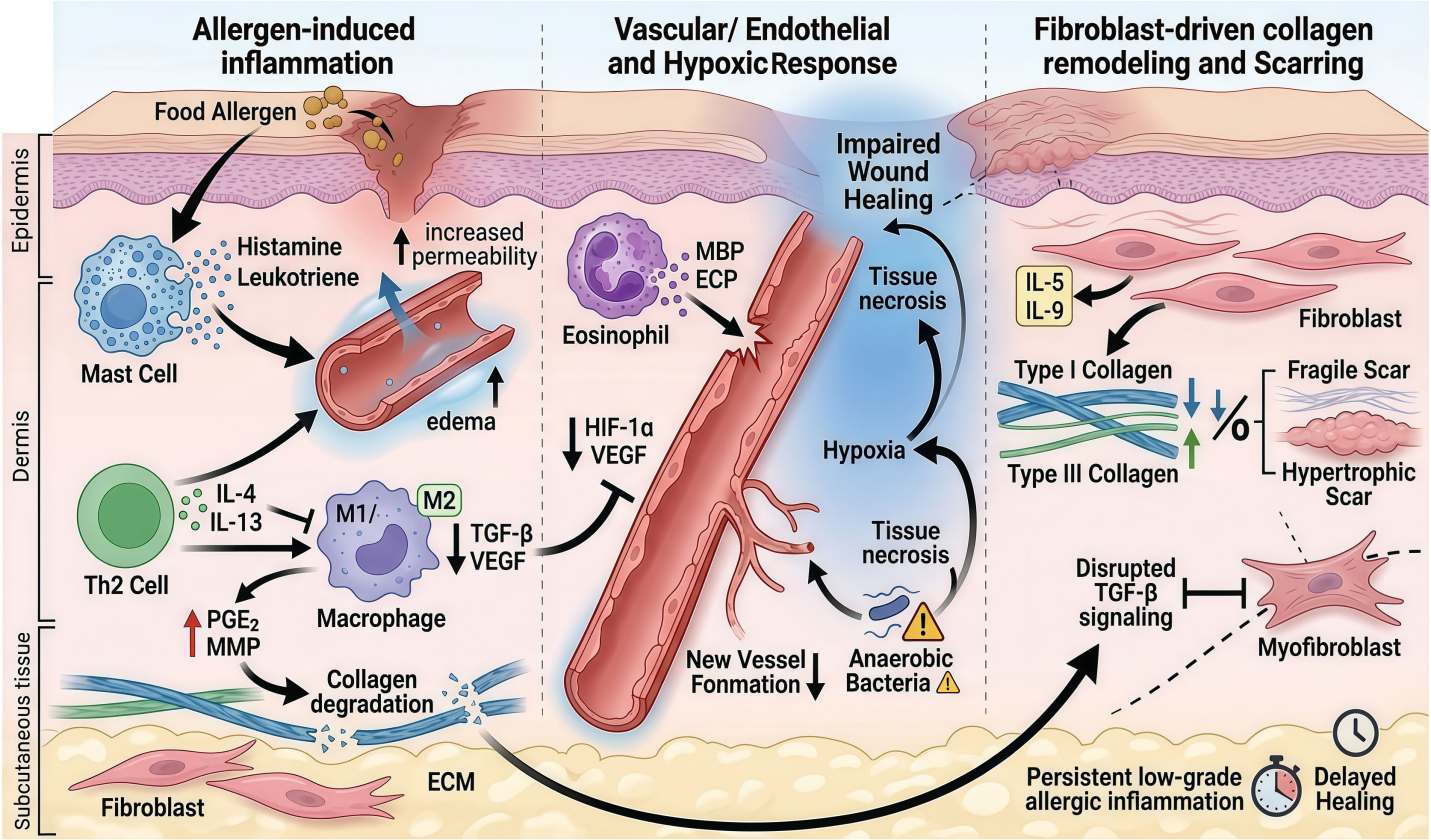
Proposed mechanisms linking food allergen exposure to impaired postoperative wound healing. This schematic illustrates how food allergen exposure may disrupt postoperative wound repair through three major biological processes: inflammatory response, angiogenesis, and tissue remodeling. **(A)** Allergen-induced inflammatory response: IgE-mediated mast cell activation releases histamine and leukotrienes, increasing vascular permeability and promoting edema formation. Sustained type 2 inflammation may dysregulate macrophage polarization and reparative capacity, potentially altering the production of pro-healing factors such as TGF-β and VEGF. Concurrently, enhanced matrix metalloproteinase (MMP) activity promotes excessive extracellular matrix (ECM) degradation. **(B)** Impaired angiogenesis and tissue hypoxia: Activated eosinophils secrete cytotoxic mediators, including major basic protein (MBP) and eosinophil cationic protein (ECP), which may compromise endothelial integrity and vascular repair. Chronic inflammatory stress may impair HIF-1α/VEGF-mediated angiogenic responses, contributing to insufficient neovascularization, tissue hypoxia, impaired antimicrobial defense, and increased infection susceptibility. **(C)** Abnormal collagen remodeling and scar formation: Type 2 inflammatory signaling may alter fibroblast function and impair TGF-β-dependent myofibroblast differentiation, disrupting the normal transition from type III to type I collagen during scar maturation. These alterations may reduce wound contraction, disturb collagen remodeling, and contribute to pathological scar formation. Overall, food allergen exposure may establish a sustained inflammatory wound microenvironment that interferes with angiogenesis, extracellular matrix remodeling, and tissue repair, potentially resulting in delayed postoperative wound healing and related complications.

Finally, the normal progression of wound healing from the proliferative phase (characterized by granulation tissue formation) to the maturation/remodeling phase (characterized by collagen reorganization and scar tissue refinement) is a tightly regulated process requiring the resolution of inflammation. A chronic, low-grade allergic inflammation, even below the threshold of acute symptoms, can persist within the wound bed for an extended period. The constant influx of inflammatory cells, particularly eosinophils and Th2 lymphocytes, and the sustained presence of their cytokines and mediators (e.g., IL-4, IL-13, IL-5, histamine) create a persistent feedback loop that prevents the normal signaling for remodeling (71). The remodeling phase involves the gradual degradation of disorganized collagen by MMPs and the synthesis of new, aligned collagen fibers by fibroblasts, a process that increases the scar’s tensile strength over time. The inflammatory mediators of the allergic response may push the system toward a chronic pro-inflammatory state that may favor active degradation (high MMP activity) over organized synthesis and alignment (low new collagen synthesis with proper crosslinking) (91). This “stalled” environment prevents the transition to the remodeling phase, leading to a prolonged state of healing (92). Clinically, this manifests as a wound that remains friable, red, and inflamed long after it should have stabilized. The protracted healing time increases the risk of further complications, including infection and poor aesthetic outcome. Ultimately, the final scar is likely to be of poor quality: either unstable and prone to breakdown or thickened and contracted, unable to achieve the necessary biomechanical properties for a durable surgical result. The proposed pathways through which food allergen exposure may impair postoperative wound healing are illustrated in **Figure 2**. 

To facilitate a structured understanding of the proposed mechanisms, a summary of the key biological pathways, supporting evidence, and their potential relevance to wound healing is provided in **Table 1**.

**Table 1:** Proposed mechanisms linking food allergen exposure to impaired postoperative wound healing and their supporting evidence.

Mechanistic category	Proposed mechanism	Representative supporting studies	Evidence type	Potential clinical implications
Allergen-induced inflammatory microenvironment disruption	IgE-mediated mast cell activation induces degranulation and releases histamine and leukotrienes, increasing vascular permeability, promoting edema, and potentially impairing the wound microenvironment.	Studies demonstrating mast cell activation, histamine-mediated vascular permeability, and edema-associated impairment of tissue oxygen diffusion and repair (54–60)	Experimental/Indirect mechanistic	Identification of patients with allergic susceptibility and perioperative strategies to control excessive inflammatory edema
	Sustained type 2 cytokine signaling (IL-4 and IL-13) dysregulates macrophage polarization and reparative function, reducing production of pro-healing growth factors including TGF-β and VEGF.	Studies on macrophage phenotype regulation, reparative macrophage function, and growth factor-mediated wound repair (61–66)	Experimental/Indirect mechanistic	Modulation of type 2 inflammatory pathways to restore reparative macrophage activity and improve wound progression
	Allergic inflammatory signaling enhances matrix metalloproteinase (MMP) activity, particularly MMP-2 and MMP-9, resulting in excessive extracellular matrix (ECM) degradation and impaired tissue remodeling.	Studies investigating MMP regulation and elevated protease activity in chronic wounds (67–69)	Experimental/Indirect mechanistic	Monitoring excessive ECM degradation and identifying wounds at risk of delayed healing
Defective angiogenesis and persistent tissue hypoxia	Activated eosinophils release cytotoxic mediators, including major basic protein (MBP) and eosinophil cationic protein (ECP), which may compromise endothelial integrity and vascular remodeling capacity.	Studies demonstrating eosinophil-associated endothelial injury and vascular dysfunction in allergic inflammatory conditions (72)	Experimental/Indirect mechanistic	Early recognition of patients with potential vascular repair impairment
	Persistent allergic inflammation may disrupt HIF-1α/VEGF-dependent angiogenic responses, limiting endothelial proliferation, migration, and new vessel formation.	Studies demonstrating the role of HIF-1α stabilization and VEGF signaling in wound angiogenesis and inflammatory regulation of hypoxia responses (73–76)	Experimental/Indirect mechanistic	Restoration of tissue oxygenation and angiogenic signaling may improve wound repair outcomes
	Sustained tissue hypoxia impairs fibroblast and keratinocyte function and weakens oxygen-dependent antimicrobial defense, promoting infection and establishing a hypoxia–infection vicious cycle.	Studies on oxygen-dependent wound repair, neutrophil antimicrobial function, and chronic wound infection susceptibility (77–81)	Experimental/Clinical	Early identification of hypoxic wounds and interventions to interrupt the hypoxia–infection vicious cycle
Dysregulated collagen deposition and pathological scar remodeling	Type 2 cytokine signaling (IL-5, IL-9, and IL-13) alters fibroblast activity and disrupts physiological type III-to-type I collagen transition, resulting in collagen I/III imbalance and a mechanically fragile wound matrix.	Studies investigating type 2 immunity, fibroblast activation, fibrosis, and collagen remodeling (82–86)	Experimental/Indirect mechanistic	Prevention of mechanically weak scars and excessive fibrotic scar formation
	Altered TGF-β signaling and impaired myofibroblast differentiation disrupt wound contraction, resulting in poor wound edge approximation and increased risk of wound dehiscence.	Studies on TGF-β regulation, myofibroblast biology, and wound contractility (86, 88, 89)	Experimental/Clinical/Indirect mechanistic	Improving wound closure and reducing postoperative wound separation
	Persistent allergic inflammation may delay the transition from proliferation to remodeling by maintaining inflammatory signaling and disturbing the balance between collagen synthesis and degradation.	Studies on type 2 immunity, allergic inflammation, fibrosis, and impaired wound remodeling (70, 90, 91)	Experimental/Indirect mechanistic	Promoting timely scar maturation and reducing progression toward chronic wound states

The mechanisms outlined in this table are primarily derived from experimental studies and indirect clinical evidence; however, robust clinical data directly linking food allergen exposure to postoperative wound healing outcomes in patients undergoing cancer surgery remain limited.

These mechanistic pathways provide a theoretical basis for exploring their potential clinical implications in postoperative wound healing, which are further discussed below.

## 5 Clinical risk factors and high-risk patient identification

### 5.1 Preoperative allergy history and immune status assessment

Patients with a documented history of food allergies face a significantly elevated risk of allergen exposure during the postoperative period, which can complicate wound healing and overall recovery. This increased risk is not limited to those with known allergies, as previously asymptomatic individuals may develop *de novo* sensitization due to the profound immunological remodeling that occurs in response to surgical stress, anesthesia, and underlying malignancy. The surgical trauma itself is thought to contribute to a systemic inflammatory response, characterized by the release of cytokines and acute-phase proteins, which can alter the balance of T-helper cell subsets and potentially break pre-existing oral tolerance to dietary antigens. Furthermore, the perioperative administration of drugs, blood products, and nutritional supplements can serve as novel allergen sources, triggering immune responses in patients with no prior history of allergic reactions. For instance, a case report documented a severe, life-threatening anaphylactic reaction to Patent Blue V dye during breast cancer surgery in a patient with no previous exposure to the dye, highlighting the unpredictable nature of perioperative hypersensitivity (93). This underscores the necessity of a thorough preoperative evaluation that goes beyond a simple history of allergic reactions. The role of preoperative biomarkers is critical in this context. Assessing baseline immune-inflammatory status through composite indices, such as the pan-immune-inflammation value (PIV), which integrates platelet, neutrophil, monocyte, and lymphocyte counts, can provide valuable information regarding systemic inflammatory status and postoperative risk stratification (94). A study on thyroid cancer patients demonstrated that a high preoperative PIV was significantly associated with more aggressive pathological features and was an independent risk factor for poor surgical prognosis, including shorter disease-free survival (95, 96). This suggests that heightened systemic inflammation and immune dysregulation prior to surgery may contribute to an increased risk of postoperative complications, potentially including allergic or hypersensitivity events. Therefore, incorporating such immune-inflammatory markers into the preoperative assessment may assist in overall postoperative risk stratification (97). A comprehensive baseline evaluation should also include the measurement of serum total IgE and specific IgE levels against common food allergens (e.g., milk, egg, soy, peanut, wheat) as well as latex, which is a well-recognized perioperative allergen. Skin prick testing can also be performed for *in-vivo* confirmation, although this should be conducted with caution in patients with a history of severe anaphylaxis or dermographism. This dual approach of serological and skin testing provides a more complete picture of the patient’s atopic status and identifies potential triggers before they are encountered postoperatively. Moreover, the assessment of immune function is paramount. Cancer-associated immune dysfunction may modify allergic immune responses in ways that are not uniformly enhancing. Although alterations in Th2-related pathways have been described in some malignancies and may be associated with altered IgE-related immune profiles, tumor-associated immunosuppression may also impair immune effector functions and attenuate clinical allergic reactivity in certain patients (98). Indeed, cancer patients have been reported to exhibit a lower prevalence of clinically observed allergic manifestations than healthy controls, accompanied by reduced IL-4/IL-17 levels and elevated TGF-β expression (98). Therefore, the relationship between cancer status and allergic susceptibility remains complex and may vary according to tumor type and individual immune status (98). In this context, comprehensive evaluation of immune-inflammatory parameters, including the pan-immune-inflammation value (PIV) (platelet, neutrophil, monocyte, and lymphocyte counts), may inform assessment of systemic inflammatory status and postoperative risk (97). International consensus on perioperative allergy management emphasizes systematic diagnostic approaches and individualized assessment based on clinical suspicion (99). However, specific biomarkers validated for predicting food allergen-related complications after cancer surgery have not been established. Thus, a meticulous pre-assessment of their lymphocyte subsets and overall immune competence is essential for tailoring perioperative management. The Arbeitsgemeinschaft der Wissenschaftlichen Medizinischen Fachgesellschaften mold guideline for medical clinical diagnostics of indoor mold exposure, while focused on a different allergen, emphasizes the importance of systematic diagnostic approaches based on clinical suspicion and individualized testing, a principle that can be directly applied to food allergen assessment in surgical patients (100). In summary, a robust preoperative workup should encompass a detailed allergy history, quantification of baseline IgE levels, specific IgE testing against likely allergens associated with intraoperative and postoperative exposures, and a thorough evaluation of the patient’s immunological health. This comprehensive assessment serves as the foundation for developing a personalized prevention strategy, guiding clinical decisions regarding perioperative nutrition and medication, and establishing a baseline against which any postoperative adverse events can be measured.

### 5.2 Sources of allergens in postoperative nutritional support

Following major oncological or other complex surgeries, enteral nutrition (EN) is a cornerstone of postoperative care, especially in patients unable to meet their nutritional requirements orally. However, some EN formulations contain intact or partially hydrolyzed cow’s milk proteins and other potential allergenic components, which may represent clinically relevant allergen sources in susceptible individuals (101). The primary protein sources in many of these formulas include cow’s milk-derived proteins (such as casein and whey), which represent common food allergen sources (101). For a patient with known or subclinical sensitivity to these proteins, the early initiation of postoperative EN can result in continuous exposure to dietary proteins during a vulnerable postoperative period. The post-surgical state is characterized by increased intestinal permeability (the “leaky gut” phenomenon), which may facilitate exposure of immune cells to luminal antigens and influence postoperative immune response (51). This is particularly concerning in the immediate postoperative period when the gut mucosa is healing and its barrier function is compromised (51). Even in patients without a preoperative diagnosis of food allergy, postoperative immune perturbation and exposure to dietary proteins may contribute to the development of new-onset food allergy-like manifestations in susceptible individuals (102). The clinical consequences of enteral nutrition-related adverse reactions may range from gastrointestinal symptoms, such as diarrhea or gastrointestinal intolerance, which may be overlooked or attributed to postoperative gastrointestinal dysfunction (103). In susceptible individuals with food allergy, exposure to relevant allergens may result in hypersensitivity manifestations, including urticaria, angioedema, and anaphylaxis (104). Beyond standard EN, patients who are transitioning to oral intake are often encouraged to consume calorically dense, soft foods like eggs (custard, scrambled eggs), dairy products (yogurt, puddings), and nut butters (peanut butter, almond butter). These represent common and potent allergen sources. If a patient’s postoperative diet is not carefully curated based on their preoperative allergy assessment, these “safe” and “nutritious” foods may trigger allergic reactions that could potentially interfere with postoperative recovery and wound healing. The risk is heightened in hospital settings where standardized menus are used and individual patient allergies may be overlooked or insufficiently communicated among the care team. A critical gap in patient safety lies within hospital food service management. The lack of standardized, visible, and actionable allergen labeling on patient meal trays, enteral formula packets, and hospital-supplied snacks is a persistent problem (105, 106). Protocol failures, such as the failure to cross-reference a patient’s allergy history with the ingredients of a provided meal or formula, represent a significant preventable cause of adverse events. For example, a patient with a confirmed soy allergy might inadvertently receive a soy-based protein supplement for postoperative healing. The establishment of a robust, multi-step screening and verification process is essential. This should include: 1) Documentation of allergies in a highly visible and accessible location in the patient’s medical record (e.g., an allergy “hard-stop” in the electronic health record). 2) Automatic system alerts for the pharmacy and dietary departments when a known allergen is ordered. 3) Mandatory, real-time verification by the nursing staff before administering any EN formula or meal, comparing the ingredient list against the patient’s documented allergies. 4) Inclusion of a clinical dietitian in the care team to prescribe and tailor the nutritional regimen, selecting specialized elemental or amino acid-based formulas when necessary to avoid intact allergenic proteins. This systematic approach is consistent with broader principles of standardized, evidence-based exposure assessment and risk management described in environmental allergen guidelines (100). By moving from a reactive approach (treating a reaction after it occurs) to a proactive one (preventing exposure), the incidence of allergen-induced complications during the critical postoperative healing phase can be significantly reduced, thereby promoting optimal wound healing and faster recovery.

### 5.3 Warning signs of poor wound healing

Postoperative wound healing is a complex, highly orchestrated process; any disruption can lead to significant morbidity. While SSIs are the most common concern, immune-mediated inflammatory reactions to allergens represent a less recognized potential contributor to delayed or impaired healing (107). To facilitate clinical recognition, the major warning signs of impaired wound healing potentially associated with allergic reactions and their clinical implications are summarized in **Table 2**. When an allergen is systemically or locally introduced in a sensitized patient, an inflammatory cascade is triggered.

**Table 2:** Warning signs of impaired wound healing potentially associated with allergic reactions after surgery.

Category	Warning signs	Clinical interpretation
Local wound findings	Persistent or disproportionate erythema, marked edema, pruritus around the wound, recurrent serous or serosanguinous non-purulent exudate, delayed epithelialization	These findings may indicate immune-mediated inflammation but may overlap with infection, ischemia, contact dermatitis, hematoma, seroma, or mechanical tension. An allergic etiology should be considered when wound abnormalities are disproportionate or temporally associated with a suspected exposure.
Timing of reaction	Rapid onset within minutes to 2 hours after exposure to food allergens, enteral formulas, drugs, latex, dyes, or antiseptics; delayed onset after 24–48 hours or several days	Rapid onset is suggestive of IgE-mediated hypersensitivity, whereas delayed manifestations may reflect T-cell-mediated or non-IgE inflammatory responses. Timing should be interpreted together with exposure history.
Systemic manifestations	Urticaria, diffuse pruritic rash, angioedema, wheezing, dyspnea, stridor, hypoxia, hypotension, nausea, vomiting, diarrhea, or abdominal cramping	Systemic manifestations increase suspicion of allergic reactions, particularly when accompanied by local wound abnormalities. Severe respiratory or cardiovascular symptoms require urgent evaluation for anaphylaxis.
Laboratory indicators	Elevated serum tryptase during acute systemic reactions, increased eosinophil count, and positive allergen-specific IgE or skin testing when clinically indicated	Serum tryptase supports mast-cell activation when measured at appropriate time points. Eosinophilia and IgE-based tests provide supportive rather than definitive evidence and should be interpreted with clinical findings.
Clinical assessment and response	Review recent allergen exposures, evaluate enteral formulas and dietary ingredients, assess medications and perioperative materials, exclude infection or ischemia, and consider allergy/immunology consultation when appropriate	Management should focus on identifying and removing suspected triggers, initiating appropriate treatment for hypersensitivity reactions, and monitoring wound progression.

These markers remain investigational and are not recommended for routine clinical use.

The first warning signs often involve the wound itself. Clinicians should maintain a high index of suspicion when a wound exhibits persistent, disproportionate redness (erythema) that extends beyond the expected inflammatory phase (first 5–7 days), marked swelling (edema) of the surrounding tissue, and the production of a serous or serosanguinous exudate that is not purulent. The wound may demonstrate delayed or absent epithelialization, meaning the edges fail to migrate across the wound bed, leading to a “stalled” healing phase. This can be mistaken for an indolent infection or poor vascular supply, leading to unnecessary wound cultures, biopsies, or debridement, whereas the underlying cause is an allergic reaction. The timing of these signs is crucial. An acute IgE-mediated reaction (Type I hypersensitivity) to a food allergen, such as the milk protein in an enteral formula, can occur rapidly (minutes to two hours) after ingestion and manifest not only systemically but also locally at the wound site, enhancing vascular permeability and edema. A delayed-type hypersensitivity (Type IV), mediated by T-cells, may appear 24–48 hours or even days later, causing a more indolent, prolonged inflammatory state that impedes fibroblast activity and collagen deposition. Concurrent systemic symptoms are a critical clue that points toward an immune-mediated, rather than a purely local, cause of poor healing. The presence of a diffuse, pruritic, or urticarial rash (hives) is a strong indicator of an ongoing type I hypersensitivity reaction. Gastrointestinal complaints such as profuse diarrhea, nausea, vomiting, or cramping are common in food allergy reactions. More alarmingly, respiratory symptoms like wheezing, stridor, dyspnea, or hypoxia suggest bronchial or laryngeal edema, which can rapidly progress to life-threatening anaphylaxis. As documented in a case report of an anaphylactic reaction to Patent Blue V dye, the presentation can be atypical and initially devoid of cutaneous signs, making diagnosis challenging (93). Therefore, any unexplained systemic symptom, even in the absence of skin changes, should prompt an immediate differential diagnosis that includes allergic reaction. In the postoperative setting, these systemic signs are often misattributed to medication side effects, ileus (for abdominal symptoms), or atelectasis/pulmonary edema (for respiratory symptoms), leading to a dangerous delay in appropriate intervention. To aid in the diagnosis, specific serological markers can be measured, providing objective evidence of mast cell and basophil degranulation. An elevated serum tryptase level is the most specific biomarker of mast cell activation and can support the diagnosis of acute systemic anaphylaxis (108). A baseline level should ideally be drawn as soon as a reaction is suspected, followed by a peak level one to two hours after the onset of symptoms, and a subsequent decline. A significant increase over baseline (often defined as a 20% increase plus 2 ng/mL) is highly suggestive of mast cell activation. Additionally, an elevated absolute eosinophil count (eosinophilia) can be a marker of an ongoing allergic or atopic diathesis, though it is less specific than tryptase for acute reactions. While a single elevated eosinophil count is not diagnostic, a persistent or rising count in the context of poor wound healing and food allergen exposure is a supportive finding. These biomarkers can be particularly valuable in guiding management, especially in patients who are sedated or unable to communicate symptoms. The decision to correlate these findings with clinical presentation is paramount, as highlighted by the systematic diagnostic approach outlined in guidelines for environmental exposure assessment (100). The activation of the pan-immune system, as measured by tools like PIV, might also reveal a general state of heightened inflammatory activity that predisposes to such complications (94–97). In summary, the constellation of persistent, non-purulent wound inflammation, coupled with cutaneous, gastrointestinal, or respiratory symptoms and corroborated by elevated serum tryptase or eosinophil counts, should trigger immediate suspicion of an allergen-driven wound-healing impairment. This recognition allows for the swift identification and removal of the offending allergen, initiation of antihistamine or corticosteroid therapy as appropriate, and the prevention of further wound deterioration, thereby salvaging the healing trajectory.

## 6 Clinical management strategies and interventions

### 6.1 Preoperative allergen screening and personalized nutrition program

Prior to elective oncologic surgery, a systematic evaluation of potential allergic triggers is a critical yet often overlooked component of preoperative optimization. Given that postoperative exposure to dietary allergens can precipitate an inflammatory cascade that directly compromises wound healing, implementing a standardized protocol for preoperative food allergen screening is clinically justified. This process should encompass both IgE-mediated immediate hypersensitivity reactions and non-IgE-mediated delayed-type immune responses, as both mechanisms can exacerbate systemic inflammation or localized tissue edema. For patients with neuroendocrine neoplasms (NENs), which are known to secrete biogenic amines like histamine and serotonin, the exogenous intake of these amines through diet can directly exacerbate symptoms and metabolic disturbances, making it imperative to implement a diet that limits the main sources of such compounds, especially in patients with genetic deficiencies of enzymes like diamine oxidase (109).

Based on the screening results, the formulation of an individualized EN plan becomes the next essential step. This plan must meticulously avoid identified allergen components, prioritizing the use of extensively hydrolyzed protein formulas or elemental amino acid-based preparations to minimize antigenic exposure. This approach is particularly valuable in patients undergoing complex surgical resections, such as those for pancreatic or gastrointestinal neoplasms, where malnutrition is prevalent and the integrity of the gut barrier is compromised (110, 111). Standardized nutritional risk scores, such as the Prognostic Nutritional Index (PNI) and the Controlling Nutritional Status score, have been validated as predictors of postoperative complications and survival in various malignancies, including intraductal papillary mucinous neoplasms and pancreatic NENs, further supporting the need for a precise, data-driven nutritional strategy (110, 112). A nutrition-enhanced ERAS protocol, which includes an oral immunonutrition drink and probiotics, has demonstrated a significant reduction in postoperative complications and hospital costs for patients undergoing thoracic neoplasm resections (113). These findings support the value of structured perioperative nutritional optimization; however, this protocol did not target allergen-specific pathways, and its outcomes do not directly inform allergen-controlled nutritional strategies.

The successful execution of such a complex intervention requires a robust multidisciplinary team (MDT) framework, actively involving surgeons, clinical dietitians, and immunologists. This collaborative approach is critical not only for the initial dietary plan but also for the dynamic, ongoing adjustment of the nutritional regimen based on the patient’s evolving clinical status and tolerance. Evidence from systematic reviews in neurosurgical patients with brain neoplasms confirms that personalized nutritional counseling, delivered by a specialist team, contributes to fewer complications and more effective functional recovery postoperatively (114). Similarly, adherence to oral nutritional supplements in cancer patients is profoundly influenced by factors that can only be addressed through close team communication, such as perceived benefits, adverse reactions, and palatability, all of which require fine-tuning that a single-discipline approach cannot provide (115, 116). In the context of NENs, the relationship between nutritional status and disease progression is nuanced, involving factors like obesity, metabolic syndrome, and chronotype, all of which necessitate an integrated management plan that an MDT is uniquely positioned to deliver (111, 117). Therefore, the preoperative period is not a static window but an active, coordinated process of screening, planning, and multidisciplinary oversight designed to prime the patient for optimal surgical recovery by mitigating the risk of allergen-induced immune disruption.

### 6.2 Perioperative immune regulation as well as anti-allergy intervention

In the perioperative period, proactive pharmacological modulation of the immune response is a crucial strategy to mitigate the detrimental effects of accidental or unavoidable food allergen exposure on tissue repair. The immediate release of histamine and other vasoactive amines from mast cells and basophils can lead to localized edema, increased vascular permeability, and a pro-inflammatory state that is antagonistic to the anabolic processes of wound healing. Therefore, the judicious use of H1-antihistamines (e.g., cetirizine) and mast cell stabilizers (e.g., sodium cromoglycate) can be considered to dampen this acute inflammatory burst. This approach aligns with the management of conditions like allergic rhinitis, where antihistamines are first-line therapy to control mucosal inflammation; in the surgical context, they serve to prevent a similar, albeit localized, inflammatory response that could impair wound strength and lead to dehiscence (118, 119). For patients with NENs, who may have elevated levels of endogenous histamine, the use of serotonin synthesis inhibitors alongside antihistamines has been discussed as a means to control symptoms of carcinoid syndrome, indicating a precedent for managing biogenic amine-related inflammation pharmacologically (109).

For high-risk patients, defined as those with a robust history of anaphylaxis or multiple food sensitivities, the short-term, low-dose administration of systemic corticosteroids in the immediate preoperative and early postoperative phase may be necessary to stabilize the overall immune system. This intervention is a delicate balance, as while corticosteroids potently suppress the Th2-driven allergic response and reduce vascular permeability, they must be weighed against the increased risk of infectious complications and impaired wound fibroblast activity. The decision to use such agents should be guided by a careful risk-benefit assessment within a MDT framework (120). Similar considerations are reflected in the management of rare, complex conditions such as eosinophilic granulomatosis with polyangiitis, where systemic corticosteroids are a mainstay of therapy but require meticulous anesthetic planning and infection surveillance. This pharmacological strategy aims not to completely suppress the immune system, but rather to attenuate an excessive, maladaptive allergic response that could derail the healing process.

A complementary, non-pharmacological approach to perioperative immune modulation involves the use of probiotics, specifically Lactobacillus and Bifidobacterium strains. The gut microbiota plays a fundamental role in shaping systemic immune responses, including the development of oral tolerance to dietary antigens. Supplementation with these beneficial microbes can help re-establish a healthy gut ecosystem, which is often disrupted by surgery, stress, and antibiotic use, thereby promoting a tolerogenic immune environment. The gut microbiota is now recognized as a key mediator in several processes crucial for surgical recovery, including angiogenesis and inflammation (121). In cancer patients, probiotics may influence tumor progression and treatment response by modulating the gut microbiome (122). The NUTRIENT trial, which investigated a Mediterranean diet intervention in myeloproliferative neoplasm patients, showed that dietary interventions are feasible and can be further developed to incorporate probiotics as a component of care for chronic clonal hematologic conditions (123). Furthermore, a high-fiber diet may potentially affect the production of beneficial short-chain fatty acids by gut bacteria, which have potent anti-inflammatory effects and can enhance wound healing, while a high-choline diet can lead to the production of pro-inflammatory metabolites like trimethylamine N-oxide, which promote cancer progression and systemic inflammation (121, 122). Thus, perioperative administration of probiotics, combined with a prebiotic-rich diet, provides a low-risk, multifaceted intervention to support immune homeostasis and directly counterbalance the pro-inflammatory consequences of inadvertent allergen exposure.

### 6.3 Applications of wound monitoring technologies

Advancements in wound monitoring technology now offer the ability to detect the earliest signs of allergic inflammation directly at the healing site, providing a dynamic feedback loop that can guide clinical decision-making in real-time. Traditional assessment of wound healing relies on visual inspection, which is subjective and cannot detect biochemical changes until they manifest as visible erythema, edema, or exudate. The systematic analysis of wound exudate for specific inflammatory and allergic mediators offers a more objective and sensitive approach. Elevated local concentrations of Th2-associated cytokines, such as IL-4 and IL-13, or the detection of histamine within the wound microenvironment, have been proposed as potential indicators of a local allergic reaction before it becomes clinically apparent. The diagnostic utility of this approach remains exploratory and has not been validated in prospective clinical studies. This early warning allows for prompt intervention, such as the application of topical corticosteroids or systemic antihistamines, to mitigate damage to the nascent granulation tissue. This concept of biomarker-driven monitoring is analogous to assessing nutritional status and systemic inflammation in conditions like gastroenteropancreatic NENs, where indicators such as chromogranin A and 5-hydroxyindolacetic acid (5-HIAA) guide treatment (109), and where PNI is used to predict progression (110). Similarly, for patients with brain neoplasms, the Monitoring of inflammatory markers like IL-6 and TNF-α in conjunction with nutritional parameters is associated with better outcomes, illustrating a broader trend toward objective biological surveillance (114).

The next frontier in this field is the development and deployment of novel sensor technologies capable of continuous, non-invasive monitoring of the wound environment. These smart dressings or implantable sensors can detect subtle changes in local parameters such as pH and temperature, which are hallmarks of acute inflammation. A decrease in wound pH is often associated with bacterial colonization and ischemia, while a localized increase in temperature is a classic sign of the inflammatory response, including that triggered by an allergic reaction. Real-time alerts generated by these sensors can empower clinical staff to intervene at the very onset of an inflammatory event, potentially preventing the rapid progression of wound breakdown. This technology is particularly relevant for complex or chronic wounds, where persistent inflammation is a major barrier to healing. For instance, patients with severe stomal retraction and moisture-associated skin damage, a condition often exacerbated by chemical irritation and potential allergic contact dermatitis, have been proposed as a population that could benefit from such monitoring strategies to guide wound and dietary management. The clinical utility of this approach remains hypothetical and has not been validated in this specific context (124). Dietary patterns and hydration habits have been suggested to be associated with wound-healing outcomes in chronic ulcer populations (125). One possible explanation is that immune alterations affecting tissue repair may also influence local responses to dietary antigens; however, this hypothesis has not yet been directly evaluated in clinical studies. The integration of these monitoring technologies into a comprehensive clinical pathway will enable a truly personalized and proactive approach to postoperative care, shifting from reactive treatment of established wound complications to early, data-driven prevention.

## 7 Future research directions and challenges

### 7.1 Targeted therapy for immune tolerance reconstruction

The concept of restoring immune tolerance to specific antigens is a cornerstone for managing allergic responses in the perioperative and postoperative periods in cancer patients. One of the most clinically advanced strategies for inducing such tolerance is oral immunotherapy (OIT), which involves the controlled, gradual introduction of an allergen to the oral mucosa to desensitize the patient. This approach has been recommended for reducing the burden of strict allergen avoidance in patients with persistent food allergies, such as those to hen’s eggs or cow’s milk, by inducing desensitization and potentially long-term immune tolerance (126). The success of OIT relies on shifting the immune response from a pro-allergic, IgE-dominated profile to one characterized by tolerance. Mechanistically, this involves the induction of Tregs and the production of blocking antibodies, particularly IgG4, which can compete with IgE for allergen binding and inhibit mast cell degranulation (127). Specifically, during the early phase of peanut OIT, single-cell RNA sequencing of Tregs has revealed dynamic changes in gene expression, including the upregulation of markers like OX40R and GITR in memory gamma delta regulatory T cells (gdTregs), suggesting these cells contribute to the tolerogenic effect and may serve as an early marker of desensitization (128). Given the prolonged escalation period required to achieve maintenance dosing, oral immunotherapy is unlikely to be feasible as a routine short-term preoperative intervention. Its potential relevance may instead be limited to selected patients with pre-existing food allergies undergoing long-term treatment planning.

Beyond OIT, identifying the specific dietary antigens that normally elicit tolerance is crucial for designing targeted immunotherapies. Recent research has identified that epitopes from seed storage proteins, such as the maize protein alpha-zein, are natural targets for murine intestinal Treg cells (129). Zein-specific regulatory T cells represent a relatively small but measurable subset of the peripheral Treg population, accounting for approximately 2% of circulating Tregs in the reported study (129). This discovery highlights that oral tolerance to common dietary components is an active, Treg-mediated process. In the context of cancer patients who may have disrupted gut and immune homeostasis, this natural tolerance could be compromised, leading to an aberrant response to these otherwise harmless antigens. Targeted preconditioning that reinforces this specific Treg-mediated tolerance to relevant dietary allergens, perhaps mirroring the natural process of tolerance development, could be a novel and highly specific approach to prevent postoperative allergic dysregulation. Engineered platforms that mimic the natural delivery and presentation of such antigens could further enhance this strategy.

An advanced therapeutic approach involves the direct enhancement of the Treg population and its suppressive functions. Although IL-10 and TGF-β are central mediators of immune tolerance, systemic administration of these cytokines remains challenging due to potential off-target effects, and current evidence does not support their direct use as a routine therapeutic strategy. Future approaches may instead focus on localized delivery systems, targeted immune modulation, or strategies designed to enhance endogenous regulatory pathways rather than direct systemic cytokine administration. Mechanistically, IL-10 and TGF-β play critical role in promoting an anti-inflammatory milieu essential for wound healing. For instance, in the context of diabetic wound healing, the failure of macrophages to transition to an anti-inflammatory M2 phenotype is a key pathological feature. The combined induction of endotoxin tolerance and M2-like polarization was associated with a generalized reduction in cytokine production, affecting both pro-inflammatory mediators such as TNF-α and IL-6 and anti-inflammatory cytokines including IL-10 and TGF-β (130). Similarly, restoring immune homeostasis and promoting regenerative phenotypic transitions are critical for effective tissue repair. The agonistic activation of the nuclear receptor LRH-1/NR5A2 has been proposed to promote phenotypic changes in pancreatic cells (131), providing a conceptual example of how inflammation resolution may be coupled with tissue regeneration. In the perioperative setting, a targeted approach to bolster Treg activity and promote M2 macrophage polarization could counteract the inflammatory insult of surgery and any coincidental allergen exposure, thereby protecting the delicate process of wound healing.

Finally, nanotechnology offers a precise platform for targeted immunomodulation. Biodegradable nanoparticles can be engineered to deliver specific antigenic peptides directly to the immune system to induce tolerance rather than immunity. A groundbreaking study in a murine model of alpha-gal syndrome demonstrated that prophylactic administration of nanoparticles encapsulating the α-gal oligosaccharide could blunt the production of Th2 cytokines and reduce α-gal-specific IgE production, effectively augmenting immune tolerance (132). This “Trojan horse” approach, where a nanoparticle co-opts immune pathways to deliver a tolerogenic signal, is highly promising. In the context of cancer surgery, such nanocarriers could be loaded with peptides from common food allergens that a patient might be exposed to postoperatively. This would allow for a controlled, targeted induction of tolerance without the risks associated with systemic immunosuppression. The ability to design these platforms to be microenvironment-responsive, for instance, activating only at the site of the surgical wound where the pH is altered or inflammation is highest, could further enhance their specificity and safety (133). By harnessing these advanced delivery systems, it may be possible to create a state of “localized tolerance”, protecting the healing wound from the detrimental effects of an allergic response while leaving the systemic immune system intact to fight any residual cancer cells.

### 7.2 Biomarkers and prediction models

The ability to predict which cancer patients are at the highest risk for developing postoperative allergic sensitization and subsequent wound healing complications is paramount for personalized intervention. This requires the identification of robust, quantifiable biomarkers that can reflect the shift from a state of tolerance to one of allergic sensitization. One of the most promising avenues is the analysis of the basophil activation test (BAT). BAT is an ex vivo functional assay that measures the activation of basophils (CD63 upregulation) after allergen exposure, providing a direct readout of IgE-mediated hypersensitivity (134). In AllergoOncology, BAT is emerging as a valuable tool for monitoring drug tolerance, predicting reactions, and discovering new biomarkers (134). A positive BAT to a specific food allergen before a major surgical procedure, even in a patient without a clear clinical history, could indicate subclinical sensitization that might be unmasked by the perioperative stress and immune dysregulation. Incorporating BAT into a preoperative risk profile could help identify patients who would most benefit from a tolerogenic preconditioning protocol.

Beyond functional assays like BAT, circulating molecular markers hold significant potential for early detection of immunological shifts. The serum microRNA (miRNA) profile is an attractive candidate, as miRNAs are stable in the blood and regulate multiple aspects of the immune response (135, 136). Changes in specific miRNA signatures have been linked to allergic diseases and are thought to play a role in regulating Th2 responses and Treg function (137–139). In the context of cancer and its treatment, monitoring miRNA profiles could help capture the early, subtle changes that precede a full-blown allergic reaction (135). Another powerful biomarker is the diversity of the T cell receptor repertoire, particularly within the Treg compartment (140). The establishment of food allergies is associated with an altered T cell response, and oral immunotherapy is known to induce changes in the frequency and gene expression of specific Treg populations, such as gdTregs, which are thought to contribute to the maintenance of immune homeostasis and tolerance (128, 141). A loss of Treg repertoire diversity or a shift toward a pro-inflammatory T cell clonotype might be a harbinger of impending loss of tolerance. Integrating these cellular and molecular markers could provide a high-definition view of a patient’s immune status, allowing for highly individualized risk stratification.

To translate these candidate biomarkers into a clinically useful tool, they must be integrated into robust predictive models. Clinical risk factors alone, such as age, comorbidities (e.g., obesity, hypertension), and disease status, are already known to correlate with alterations in the expression of immune-related molecules associated with inflammatory pathways (142). Furthermore, the type of surgical procedure (e.g., major orthopedic surgery vs. minor skin excision) and the specific targeted cancer therapies used (e.g., immunotherapy, which can have unique wound healing implications) are critical variables (143, 144). A modern predictive model must therefore integrate these clinical factors with the novel immunological biomarkers. Machine learning algorithms are ideally suited for this task, as they can handle the high dimensionality of such data (e.g., hundreds of miRNA levels, thousands of TCR sequences) and identify complex, non-linear interactions that simple logistic regression could miss. For instance, integrating baseline immune parameters, nutritional status (as malnutrition is a known risk factor for poor healing), and specific surgical and oncological details into an ML model could yield a highly accurate risk score. This model could then be used to guide decision-making, such as selecting patients for preoperative OIT or more intensive postoperative monitoring.

The ultimate validation of these biomarkers and models requires well-designed prospective cohort studies. The Immunophenotyping Assessment in a COVID-19 Cohort (IMPACC) study serves as an excellent example of how comprehensive, longitudinal clinical and immunological phenotyping can identify distinct disease trajectories and risk factors for adverse outcomes (145). A similar approach is urgently needed for postoperative allergic sensitization. Such a study would need to collect serial blood samples and detailed clinical data from a large, diverse cohort of cancer patients undergoing surgery. This would allow researchers to track the temporal evolution of candidate biomarkers (e.g., BAT reactivity, miRNA levels, Treg counts) before and after surgery and correlate changes with clinical endpoints, such as the development of food allergy symptoms, wound dehiscence, or infection. At present, direct evidence supporting this mechanism in postoperative cancer patients is lacking. We propose that future investigations explore whether alterations in immune tolerance, allergen responsiveness, and wound-healing dynamics may be mechanistically interconnected. This concept should currently be regarded as a theoretical framework rather than an established biological pathway. Only through such rigorous validation can these tools move from the research bench to the patient’s bedside.

### 7.3 Accumulation of multicenter clinical evidence

A critical bottleneck in translating the promising concepts of immune tolerance and allergic risk management into clinical practice is the profound lack of high-quality evidence. The vast majority of studies exploring the link between allergen exposure, immune modulation, and wound healing in the perioperative oncology setting are small, observational, and often single-center (146–148). While these studies provide crucial mechanistic insights, they are insufficient to establish a definitive causal relationship or to inform clinical guidelines. For example, while we understand the role of macrophages in wound healing and the potential for an allergic response to derail this process, the specific contribution of a postoperative allergic reaction to the failure of a surgical wound has not been robustly proven in a prospective, controlled trial (149, 150). The absence of large-scale, randomized controlled trials leaves a significant gap in our knowledge, making it difficult for clinicians to make evidence-based decisions about whether and how to screen for or prevent allergic events in this specific population. This is a critical gap, as the field of chronic wound care itself acknowledges the complexity of host-microbiome interactions and the challenge of managing infections, which are further complicated by underlying immune dysregulation (151).

To overcome this, a concerted effort to standardize research protocols is an absolute prerequisite. A major impediment to meta-analysis and cross-study comparisons is the heterogeneity in how outcomes are defined and measured. For wound healing, the assessment is often subjective, with researchers using different scales or criteria for healing time, scar quality, and the presence of infection. A unified set of endpoints must be established, likely including objective measures like wound closure time, validated scar assessment scales (e.g., the Patient and Observer Scar Assessment Scale), and standardized definitions of surgical site infections. Similarly, the recording of allergen exposure needs to be rigorous and consistent. Currently, it is often based on retrospective patient recall, which is notoriously unreliable. Future studies must implement prospective food diaries or, ideally, utilize controlled dietary protocols during the perioperative period to ensure that exposure is accurately documented and quantified. Furthermore, the methods for diagnosing an allergic reaction must be standardized, employing objective tests like the basophil activation test (BAT) for confirmation, rather than relying solely on clinical symptoms, which can be confounded by the normal inflammatory response to surgery (146, 147). Only with these standardized tools can data from multiple centers be aggregated meaningfully.

The scale and complexity of the required research mandate a collaborative, multi-disciplinary approach. No single institution or specialty can effectively answer the question of how a specific dietary protein might impact a surgical wound in a patient with a unique tumor type and treatment history. This requires a network of experts in allergy/immunology, surgical oncology, wound care, nutrition, and data science. Such a collaboration is essential for developing a comprehensive, standardized research protocol that addresses the multifaceted nature of the problem. Furthermore, the field would be greatly advanced by the establishment of an international, open-access clinical registry. This registry would collect de-identified data from patients across multiple centers, using the standardized core variables for wound healing, allergen exposure, and immune function. Such a registry would serve as a powerful engine for research. It would provide the statistical power to identify rare events, control for a wide range of confounders (e.g., type of cancer, surgical technique, use of biologics), and generate hypotheses for future studies. The power of such a big data approach has been demonstrated in other complex fields like COVID-19 research, where large collaborative efforts like the IMPACC study rapidly identified predictors of disease severity (142). A similar global repository for perioperative allergic complications could accelerate the accumulation of robust clinical evidence, enabling powerful meta-analyses and ultimately providing the definitive evidence needed to change clinical practice and improve outcomes for cancer patients.

## 8 Conclusion

The post-malignant tumor immune microenvironment undergoes profound remodeling, fundamentally altering the host’s homeostatic tolerance to food allergens. This phenomenon, while stemming from the intricate interplay between cancer immunology and mucosal immunity, has emerged as a distinct clinical concern in the context of postoperative wound healing. The relationship between allergen exposure and impaired recovery is not merely correlative but mechanistically grounded. Allergens, through the preferential activation of Th2-type immune responses, initiate a cascade of events that extend beyond typical allergic inflammation. They disrupt the tightly regulated angiogenic and fibrotic processes essential for proper wound closure. Specifically, the release of cytokines such as IL-4, IL-5, and IL-13 can suppress the recruitment and function of macrophages involved in debris clearance and tissue regeneration, while simultaneously promoting aberrant collagen deposition that predisposes patients to hypertrophic scarring or keloid formation. Moreover, the vasoactive mediators triggered during allergic responses may lead to local edema and microvascular instability, further compromising the nutrient and oxygen supply necessary for cellular regeneration. Thus, food allergen exposure in the postoperative period represents an independent and modifiable risk factor for delayed healing, persistent inflammation, and poor aesthetic outcomes.

Reconciling these research findings requires acknowledging both the robustness of the mechanistic links and the heterogeneity of patient presentations. While preclinical models have robustly elucidated the pathways through which allergens disrupt wound healing, clinical evidence remains fragmented. Individual susceptibility to allergic responses varies based on prior sensitization, genetic predisposition, tumor type, and the specific allergenic epitopes involved. Consequently, a uniform approach to perioperative management is insufficient. Instead, a nuanced, stratified strategy is essential. The clinical imperative, therefore, is to integrate preoperative screening for common food allergens—particularly in patients with known atopy or those undergoing extensive tumor resection—with personalized dietary counseling. This shifts the paradigm from reactive management of allergic complications to proactive risk mitigation. Concurrently, the concept of immune modulation during the perioperative window must be refined. Agents that selectively modulate Th2 responses without broadly suppressing immune surveillance—such as targeted biologics or specific dietary interventions—warrant rigorous investigation. Furthermore, dynamic monitoring of wound healing trajectories using biomarkers, such as serum levels of eosinophil-derived neurotoxin, may enable early detection of allergen-related delays and prompt timely intervention.

Looking toward future directions, the most compelling research frontier is the targeted restoration of immune tolerance in postoperative patients. This involves not only desensitization protocols but also the modulation of the gut-skin axis, given the critical role of gastrointestinal immune homeostasis in shaping systemic responses to allergens. Emerging strategies, such as oral immunotherapy combined with checkpoint inhibitors or pro-tolerogenic microbial therapies, hold theoretical promise but require caution, as any immunomodulation must avoid interference with cancer-specific immune responses. Additionally, the development of reliable biomarkers—whether derived from serum metabolomics, microbiome profiling, or skin transcriptomics—is paramount to stratify patients and guide individualized treatment plans. Multicenter clinical trials with standardized endpoints, including validated wound healing scales and allergic reaction monitoring, are urgently needed to provide the evidence base that is currently lacking. Future progress in this field will depend on the generation of direct clinical evidence linking postoperative food allergen exposure to wound-healing outcomes in patients undergoing cancer surgery. Prospective cohort studies are needed to determine whether perioperative allergen exposure, pre-existing food hypersensitivity, or alterations in immune tolerance influence postoperative recovery and wound-related complications. In parallel, translational investigations should further explore the interactions among allergic inflammation, immune regulation, tissue repair, and tumor-associated immune remodeling. The identification and validation of biomarkers reflecting immune tolerance status, allergic sensitization, and wound-healing capacity may facilitate risk stratification and early intervention. In addition, future clinical trials should evaluate whether individualized nutritional management strategies can improve postoperative outcomes in selected patient populations with known or suspected food hypersensitivity. Such studies will be essential for determining the clinical significance of the mechanisms proposed in this review and for establishing evidence-based recommendations.

Ultimately, the convergence of oncology and allergology necessitates a collaborative, interdisciplinary approach. The impact of this work extends beyond improving recovery rates; it underscores the systemic nature of post-cancer immune dysfunction and the interconnectedness of seemingly disparate clinical phenomena. As our understanding deepens, the goal becomes clear: not merely to avoid allergens but to strategically manage the immune environment to facilitate optimal healing and long-term quality of life for cancer survivors. By integrating mechanistic insights with patient-centered care and investing in translational research, we can transform a newly recognized clinical risk factor into a manageable component of comprehensive survivorship management. From a clinical perspective, individualized nutritional assessment and dietary management may represent an important component of perioperative care in selected cancer patients with known or suspected food hypersensitivity. Although direct evidence remains limited, consideration of allergen exposure, nutritional status, and immune function may help identify patients at increased risk of postoperative complications. Future integration of nutritional, immunological, and wound-healing assessments may facilitate more personalized perioperative management strategies and improve postoperative recovery.
